# Effect of radiation therapy on the dentin bond strength of a universal adhesive

**DOI:** 10.1186/s12903-025-06226-5

**Published:** 2025-05-27

**Authors:** Pipop Saikaew, P Hannongbua, P Jianratanajit, I Wongkertprayot, P Chaiklahan, N Srimaneekarn, A Prayongrat, P Eamsa-ard, AFMA Chowdhury, K Katekovit, S Hidehiko

**Affiliations:** 1 Department of Operative Dentistry and Endodontics, Faculty of Dentistry, Mahidol University, 6 Yothi Road, Ratchathewi, Bangkok, 10400 Thailand; 2 Faculty of Dentistry, Mahidol University, 6 Yothi Road, Ratchathewi, Bangkok, 10400 Thailand; 3 Department of Anatomy, Faculty of Dentistry, Mahidol University, 6 Yothi Road, Ratchathewi, Bangkok, 10400 Thailand; 4 Division of Radiation Oncology, Department of Radiology, Faculty of Medicine, Chulalongkorn University, King Chulalongkorn Memorial Hospital, Bangkok, 10330 Thailand; 5 Department of Operative Dentistry, College of Dental Medicine, Rangsit University, 52/347 Muang-Ake Paholyothin Road, Lakhok, 12000 Pathum Thani Thailand; 6 Department of Conservative Dentistry and Endodontics, Sapporo Dental College and Hospital, Plot 24, Sector 8, Dhaka, 1230 Bangladesh; 7 Department of Restorative Dentistry, Faculty of Dental Medicine, Hokkaido University, Kita 8, Nishi 5, Kita-ku, Sapporo, 060-080 Japan

**Keywords:** Universal adhesive, Dentin, Microtensile bond strength, Radiation

## Abstract

**Background:**

Successful restorative procedures, particularly for patients undergoing head and neck radiotherapy, rely on a robust adhesive interface. Since radiotherapy may alter dental tissues and compromise bonding, this study evaluated how the timing of radiation exposure affects the microtensile bond strength (µTBS) of a universal adhesive to dentin with different application modes.

**Methods:**

Forty-two human third molars were used in this study. Mid-coronal dentin was prepared using a low-speed saw and polished with silicon carbide paper. The samples were randomly divided into 3 groups (*n* = 6) according to the timing of radiation exposure (no radiation, control; radiation after restoration, F-RT; and radiation before restoration, RT-F). A single radiation dose of 70 Gy was administered to the samples after restoration (F-RT) and before restoration (RT-F). The samples were further divided into 2 subgroups according to the application mode of a universal adhesive (Single Bond Universal adhesive, 3 M Oral Care, St. Paul, MN, USA): etch-and-rinse (ER) or self-etch (SE) mode. After adhesive application, the resin composite was constructed and stored in distilled water at 37 °C for 24 h. Six resin-bonded teeth per group were processed for the µTBS test. The data were analyzed by two-way ANOVA followed by Duncan’s test (*p* < 0.05). Fractured surfaces were observed under a scanning electron microscope (SEM). Additional teeth were prepared for resin‒dentin interface observation (*n* = 1) and observed using an SEM.

**Results:**

The application mode of the universal adhesive had no influence on the µTBS of dentin, regardless of irradiation (*p* = 0.670). The µTBS values of the control groups were not significantly different from those of the F-RT group but were significantly higher than those of the RT-F group for both application modes. The resin‒dentin interfaces of the samples were similar among the control, F-RT and RT-F groups. More abundant and longer resin tags were observed when the universal adhesive was bonded in ER mode.

**Conclusion:**

Compared with radiation after restoration, radiation before restoration resulted in an inferior bond strength. The application mode of a universal adhesive had no effect on the bond strength.

**Clinical significance:**

Restoration prior to the radiation procedure is an advisable strategy.

## Introduction

Head and neck cancer remains a significant global health challenge, and while surgery and chemotherapy are common treatment modalities, radiotherapy has become a widely accepted approach because of its ability to selectively target malignant cells using ionizing radiation [1, 2]. In clinical practice, radiotherapy for head and neck cancer typically involves an accumulated dose ranging between 50 and 70 Gy, which is delivered in daily fractions of 2 Gy over 5 to 7 weeks [3]. This regimen is designed not only to maximize the cytotoxic effects on cancer cells but also to allow for normal tissue repair [4].

Despite its therapeutic benefits, radiotherapy impacts both malignant and healthy tissues. In the oral cavity, these effects may include salivary gland dysfunction, alterations in the oral microbiota, and hypovascularization, all of which contribute to a heightened risk of postradiation caries and other dental complications [5]. Radiotherapy also adversely affects the structural integrity of hard dental tissues. Ionizing radiation can modify the crystalline structure of enamel and dentin, leading to microcrack formation and demineralization characterized by a reduction in the calcium/phosphorus ratio. Consequently, these changes diminish the surface hardness, permeability, and overall biomechanical properties of dental tissues [6, 7].

Comprehensive oral care before radiotherapy is critical to mitigate these complications. Pretreatment dental assessments, extractions, scaling, and timely restorative procedures are essential to eliminate potential sources of infection and preserve tooth structure [5, 8]. In fact, several studies have suggested that performing restorations prior to radiation therapy can increase the bond strength between restorative materials and dental substrates [3, 9, 10]. However, in some clinical scenarios, the severity of the cancer necessitates initiating radiotherapy before all dental treatments, including restorations, are completed. Therefore, investigating how the timing of restoration—prior to versus after radiation exposure—affects adhesive performance is important. Although the impact of radiation on the bond strength of conventional etch-and-rinse adhesive systems has been documented [10, 11], limited information is available regarding the performance of modern universal adhesives under these conditions.

Universal adhesives, such as Single Bond Universal (3 M Oral Care, St. Paul, MN, USA), offer the advantage of versatility by allowing clinicians to select either etch-and-rinse or self-etch application modes [12]. Previous research has shown comparable bond strengths for these adhesives across different application methods in nonirradiated dentin [13]. However, the effect of radiation on the adhesive performance of universal systems remains underexplored. In addition, investigating the bond strength of universal adhesives in different etching modes to facilitate a more direct comparison of the effects of the etching mode and radiation timing on the bond strength while minimizing confounding variables associated with the adhesive composition is interesting. Therefore, this study aimed to investigate the influence of radiotherapy on the microtensile bond strength (µTBS) of a universal adhesive applied with different etching modes. The null hypothesis tested was that the bond strength of the universal adhesive is not affected by (1) the timing of radiation exposure relative to the restorative procedure or (2) the mode of adhesive application (etch-and-rinse versus self-etch).

## Materials and methods

### Specimen preparation

The study was performed after receiving ethical approval from the Ethics Committee (No. MU-DT/PY-IRB 2018/044. 1610). Forty-two healthy human third molars were extracted for orthodontic reasons from a hospital setting under anonymized conditions. The collected teeth were free from cracks, carious lesions or restorations. The samples were stored in 0.1% thymol after extraction and used within 6 months. Then, the apical 1/3 of the root was trimmed, and the remaining tooth was attached to an acrylic jig with cyanoacrylate glue (Model Repair 2 Blue, Dentsply-Sankin, Otahara, Japan). A cross-sectional cut was made to expose the mid-coronal dentin using a low-speed saw (Isomet, Buehler, Lake Bluff, Illinois, USA). The dentin surface was subsequently manually polished with 600-grit silicon carbide paper (TOA DCC, TOA paint, Samut Prakan, Thailand) for 1 min to standardize the smear layer [14].

**Fig. 1:**
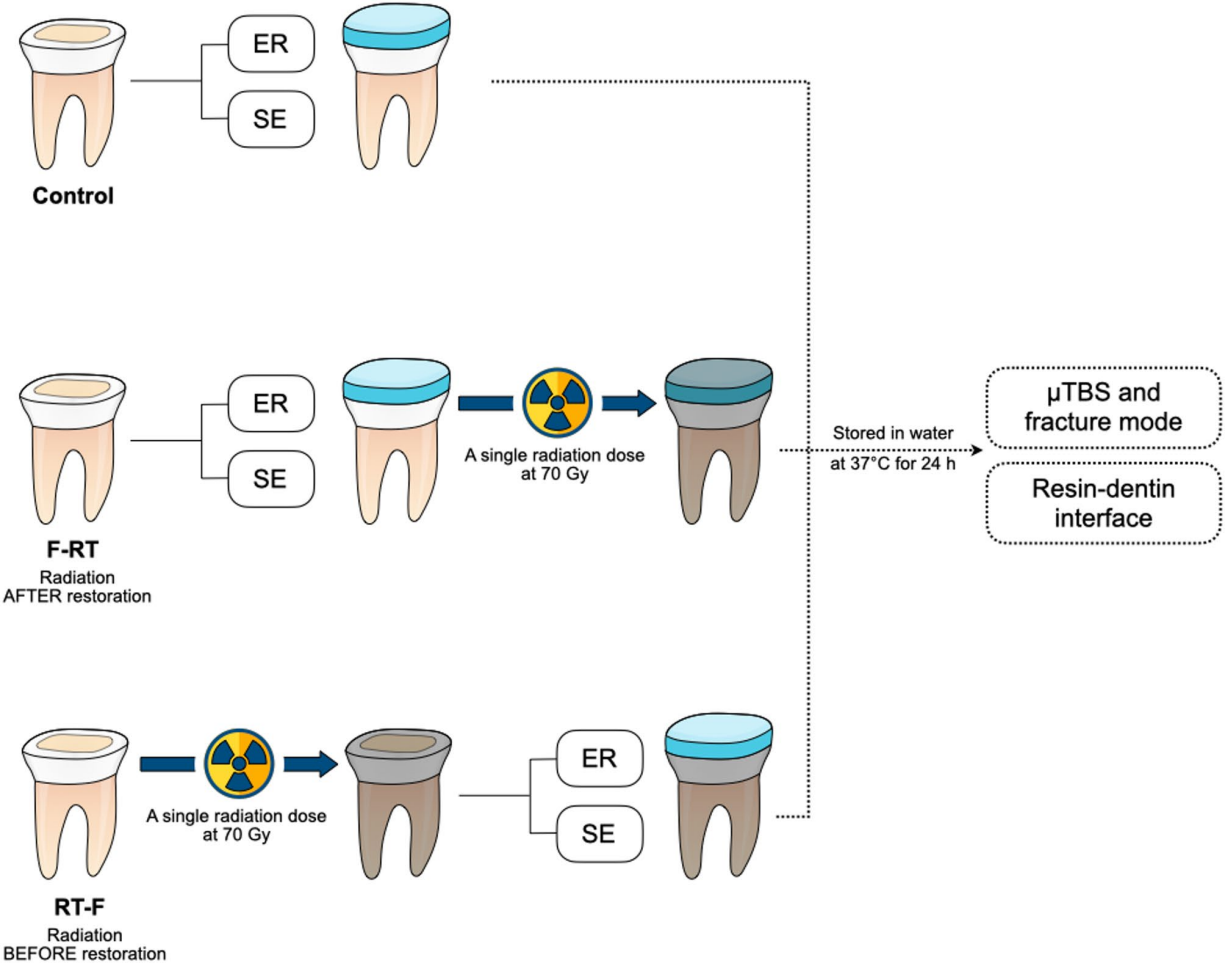
Schematic diagram for the experiment

The samples were randomly divided according to 2 variables: (1) the timing of radiation (control—no irradiation, irradiation after restoration—F-RT, and irradiation before restoration—RT-F) and (2) bonding with a universal adhesive either in etch-and-rinse mode or self-etch mode. Figure 1 illustrates the schematic diagram of the experimental setup.

**Table 1:** Material compositions and instructions of the adhesive systems used in this study

Materials	Manufacturer	Main components
Single Bond Universal Adhesive	3 M Oral care, St. Paul, MN, USA	MDP Phosphate Monomer, Dimethacrylate resins, HEMA, Vitrebond™ Copolymer, Filler, Ethanol, Water, Initiators, Silane

### Bonding procedure

The material composition is provided in Table 1. Single Bond Universal Adhesive (3M Oral Care, St. Paul, MN, USA) was applied to each tooth using either the etch-and-rinse or the self-etch mode. For the etch-and-rinse mode, Scotchbond™ universal etchant was applied to the dentin surface for 15 s, which was then rinsed with water for 30 s. An oil-free triple syringe was used to deliver the air flow, ensuring moist dentin. Single Bond Universal Adhesive was applied via a rubbing motion for 20 s using a disposable applicator. The adhesive was blown with air for 20 s to ensure optimal solvent evaporation and a uniform adhesive layer. The samples in the self-etch group were prepared in the same manner as previously described without the use of the Scotchbond™ universal etchant.

A resin composite (Filtek™ Z250,3M Oral Care) was applied in 2 increment layers, each 2 mm thick, resulting in a total thickness of 4 mm. Each layer was cured with a light-curing unit (Bluephase G2, Ivoclar Vivadent, Lichtenstein) for 20 s. A light radiometer (Bluephase^®^ meter, Ivoclar Vivadent AG, Schaan, Liechtenstein) was used to periodically measure the light intensity, ensuring that it was not less than 1,200 mW/cm^2^. The samples were subsequently stored in distilled water at 37 °C for 24 h. Teeth in the control group were further prepared for microtensile bond strength (µTBS) tests.

### Radiation protocol

The radiation protocol was performed with a single radiation dose of 70 Gy. It was conducted by a linear accelerator using 6 MV X-rays (Varian, Palo Alto, CA, USA) a dose rate of approximately 6 Gy/min and a source‒surface distance of 80 cm between the radiation cycles. The teeth were stored in distilled water during radiation to obtain a uniform radiation dose. The teeth in the F-RT group were prepared according to the bonding procedure described above, followed by radiation. The teeth in the RT-F group were subjected to radiation and then prepared according to the bonding procedure.

### Microtensile bond strength test (µTBS)

After storage in water for 24 h, the central part of the resin-bonded teeth was sectioned longitudinally into beams with a cross-sectional area of approximately 1 × 1 mm^2^, yielding 4‒9 resin‒dentin beams per tooth. The sample was mounted on a Ciucchi jig with cyanoacrylate glue (Model Repair II Blue, Dentsply-Sankin, Ohtawara, Japan) and stressed until failure using a microtensile tester (Bisco, Inc., Schaumburg, United States) at a crosshead speed of 0.5 mm per minute. The load at failure was divided by the bonding area. The microtensile bond strength was calculated in MPa. The µTBS values of the samples from the same tooth were averaged and used for the statistical analysis [14].

### Failure mode analysis

The fractured beams were mounted on an aluminum stub with a carbon double-sided tape, coated with palladium (K500X sputter coater, SPI Supplies, West Chester, PA, USA) and evaluated using a scanning electron microscope (SEM, JEOL Ltd., Tokyo, Japan). The failure mode was classified as follows: (1) adhesive failure, (2) cohesive failure within dentin, (3) cohesive failure within the resin composite and (4) mixed failure [14].

### Resin‒dentin interface observation

Six teeth were prepared in the same manner as previously described for the bond strength test. Two resin‒dentin slabs were obtained from each tooth by cutting in the buccolingual direction, with each slab being approximately 1 mm thick. The slabs were sequentially polished using silicon carbide (SiC) papers with grits of #600, #800, #1,000, and #1,200, followed by diamond paste with particle sizes of 6, 3, 1, and 0.25 μm. Each polishing step was performed for one minute, and the samples were then ultrasonicated after each polishing step for five minutes to remove residual debris. The samples were treated with 5% HCl for 30 s followed by NaOCl for 5 min. After storage in a desiccator for 24 h, the samples were coated with Pt-Pd for 150 s and observed using an SEM at ×3,000 magnification [15].

### Data analysis

The data were recorded and statistically evaluated using IBM SPSS Statistics for Windows, Version 27.0 (Armonk, NY: IBM Corp). The Shapiro‒Wilk test was performed, and the results indicated that the data were normally distributed (*p* = 0.967). Variables among groups were compared using two-way analysis of variance (ANOVA) and the Duncan test. The chi-square test was used to analyze the failure mode.

## Results

**Table 2:** Microtensile bond strength (MPa) values (means ± standard deviations) of the different experimental groups

Application mode	Microtensile Bond Strength (MPa)			
	Control	F-RT	RT-F	
Etch-and-rinse	48.24 ± 3.50 ^A^	48.71 ± 11.00 ^A^	34.74 ± 9.56 ^B^	
Self-etch	49.14 ± 7.77 ^A^	48.80 ± 10.52 ^A^	37.31 ± 4.57 ^B^	

Different capital superscript letters indicate statistically significant differences in MPa (*p* < 0.05)

No posttreatment failures (PTFs) were observed in this study. The overall values of the tested groups are shown in Table 2. Two-way ANOVA revealed no significant interaction between the tested factors (*p* = 0.933). A significant effect of the sequence of radiation was detected (*p* < 0.001), whereas the application mode of the universal adhesive had no significant effect on the dentin bond strength (*p* = 0.670).

**Fig. 2:**
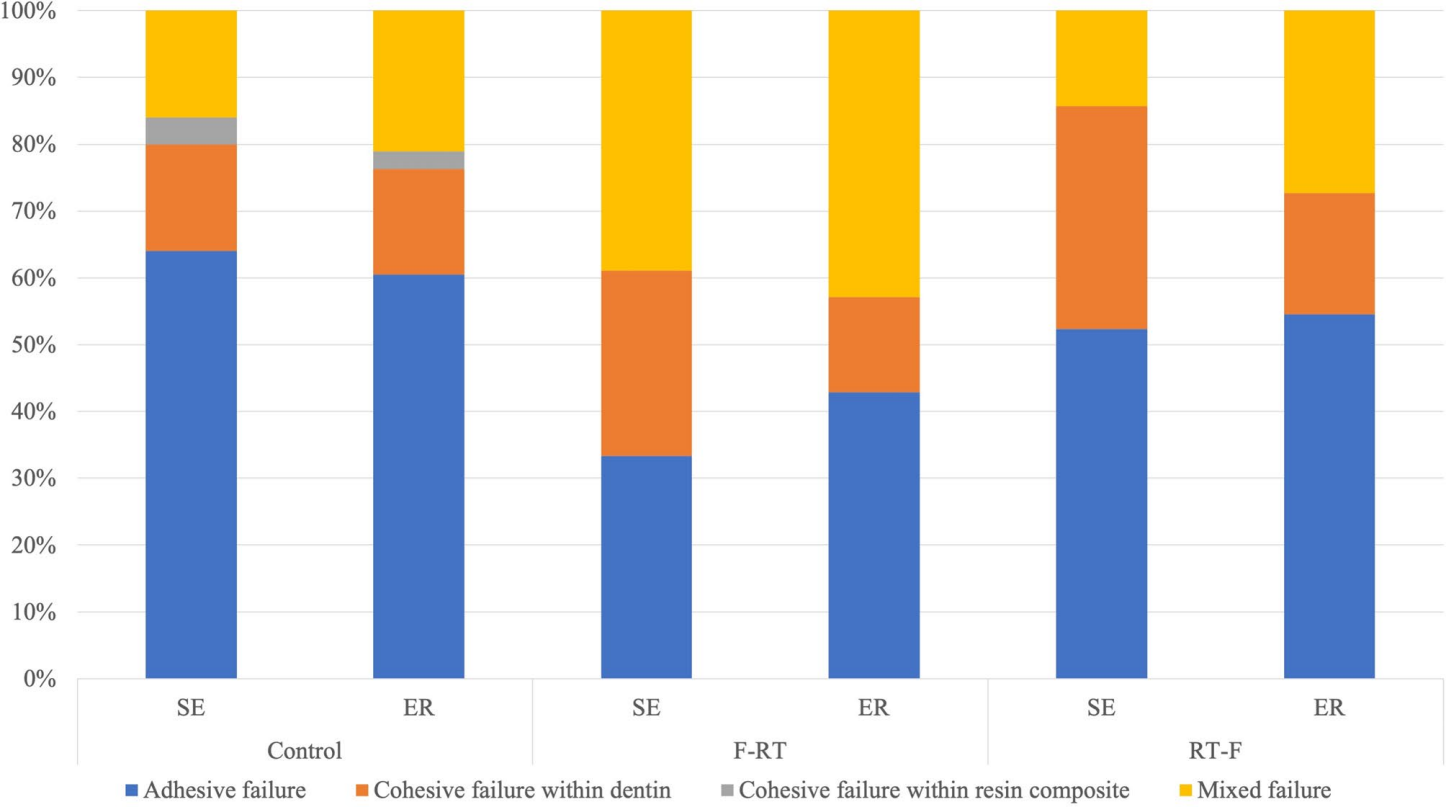
Analysis of the percentages of failure modes

Figure 2 Failure mode distributions of the experimental groups.

The analysis of the percentages of failure modes is shown in Fig. 2. The predominance of adhesive failure was observed for the control groups. The percentage of cohesive failure in dentin and mixed failure increased when the samples were restored before radiation (F-RT). Cohesive failure in the composite was detected only in the control group and not in the radiation groups. A comparison of the two application modes revealed that the self-etch mode resulted in a greater percentage of cohesive failure in dentin for both radiation groups. However, according to the chi-square test, neither factor (adhesive application mode and sequence of radiation) had a significant effect on the failure mode (*p* > 0.05).

**Fig. 3:**
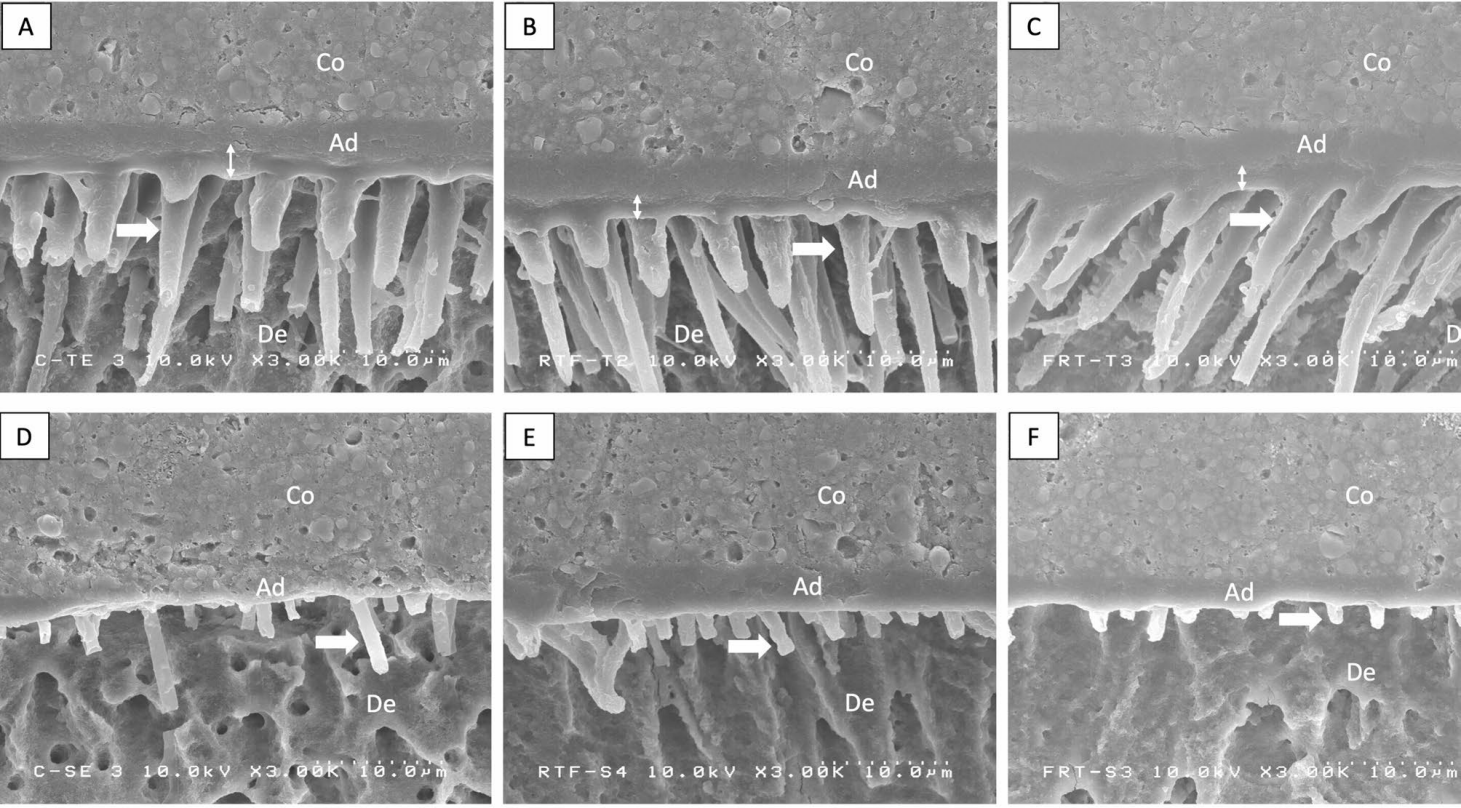
Representative images of the resin‒dentin interface of Single Bond Universal Adhesive bonded to dentin with different sequences of radiation and adhesive application modes. (**A**-**C**) Etch-and-rinse mode: (**A**) control, (**B**) RT-F—irradiation before restoration, (**C**) F-R—irradiation after restoration. (**D**-F) Self-etch mode: (**D**) control, (**E**) RT-F—irradiation before restoration, (**F**) F-RT—irradiation after restoration

Representative SEM images of the resin‒dentin interface are shown in Fig. 3. Approximately 2–4 microns of hybrid layers were observed in the ER group (Fig. 3A and C). The resin tags in the ER group were conical in shape and more abundant. However, the resin tags in the SE group were cylindrical in shape and sparsely distributed (Fig. 3D and F). No significant differences in hybrid layer thickness or resin tag formation were detected between the control group and the irradiated dentin groups.

## Discussion

Based on the findings of this study, a significant reduction in bond strength was observed when the restoration procedure was performed after radiation (*p* < 0.05); therefore, the null hypothesis asserting that the timing of radiation does not affect the bond strength of the universal adhesive was rejected. Conversely, since no significant difference in bond strength was observed between the different application modes (*p* > 0.05), the null hypothesis stating that the adhesive’s bond strength is not affected by its application mode was accepted.

In clinical situations, a fractionated dose is used for the treatment of head and neck cancer to avoid alterations in the salivary glands and soft tissues [7]. However, due to the limitations of this study, radiation was applied in nonfractionated protocols. The nonfractionated dose represents a greater amount of ionizing radiation and a less realistic clinical treatment for head and neck cancer. However, as the fractionated dose is cumulative, the final dose of radiation to the tooth substrate is similar [7]. Therefore, a single dose of 70 Gy was used in this study. In addition, a recent study reported no difference in dentin surface microhardness after exposure to a single dose and a fractionated dose [16].

The effect of the adhesive system on the bond strength of radiated dentin was investigated in previous studies [17, 18]. However, the bond strengths of two adhesives with different etching systems were compared. The different components of the adhesives might affect the bond strength. Therefore, a universal adhesive that can be applied with different etching modes was used in this study. Single Bond Universal (or Scotchbond Universal) Adhesive was selected because it was the first commercial universal adhesive [19] and has been extensively referenced in over two hundred publications in PubMed [20]. Additionally, it has the longest available clinical data, with follow-up periods extending up to five years [21, 22].

We utilized the microtensile bond strength (µTBS) test in this study because of its established reputation as a standard and versatile method for assessing bond strength [23]. The technique is well supported in the literature, with over a thousand publications available on PubMed documenting its reliability and applicability in diverse settings [24]. This widespread acceptance underpins our confidence in the use of the µTBS test as an appropriate tool for our investigation. According to the bond strength test, a significant effect of the etching mode of the universal adhesive was not detected, regardless of the dentin condition. Although the percentage of cohesive failure in dentin was higher in irradiated dentin from the SE group (Fig. 2), the difference was not statistically significant. According to the resin‒dentin interface observations, more abundant and larger resin tags were observed in the ER mode, which could be related to stronger bonding. However, the bond strengths between the ER and SE modes were similar (Table 2). This result is supported by a systematic review and meta-analysis that reported that the bond strength of a universal adhesive to dentin was not affected by the etching mode [25]. The rationale for the similar bond strengths observed between the etch-and-rinse and self-etch modes can be attributed to several factors. The incorporation of the functional monomer MDP (10-methacryloyloxydecyl dihydrogen phosphate) is crucial, as it chemically bonds with calcium in hydroxyapatite, regardless of the etching strategy [26]. Additionally, the pH of the Single Bond Universal Adhesive is optimized at 2.7, which is balanced to avoid the drawbacks associated with adhesives that have pH values that are too high or too low. Adhesives with a strong (high) pH tend to be too alkaline, resulting in minimal etching effects and, consequently, inferior bonding outcomes compared with those achieved with etch-and-rinse techniques [27, 28].

The resin composite, adhesive and dentin are three compartments in the samples. Previous studies [29–31] reported no effect of radiation on the resin composite. This result could be because the components of the resin composite, such as the organic matrix, inorganic filler and silane, are resistant to radiation. On the other hand, the destructive effects of radiation on dentin and hybridization processes have been reported [7, 11, 32]. The bond strength of the adhesives was significantly decreased when radiation was applied before the restoration procedure, which was correlated with previous studies [7, 32]. However, a study showed that the bond strength of universal adhesives could be improved if the double application technique was applied to irradiated dentin [33]. In addition, the chemical composition of dentin was altered after radiation. Radiation exposure demineralizes the inorganic and organic components of dentin, resulting in a reduction in the Ca/P weight ratio [7]. The changes in the organic component could be explained by the activation of enzymes such as metalloproteinases, which degrade collagen fibers, the main components of dentin, after radiation. Dentin structures were also disorganized, as detected by SEM [34]. Furthermore, it was hypothesized that radiation has a direct harmful effect on the hybridization process of dentin by generating reactive oxygen species (ROS), such as hydroxyl radicals, hydrogen peroxide and superoxide anions. As ROS are produced, the polymerization process of resin monomers, which normally infiltrate the pores of collagen fibrils, is inhibited [35, 36]. All of these factors negatively interfere with the adhesion process of restoration materials, resulting in a reduction in microtensile bond strength when the restoration procedure is performed after radiation [34]. On the other hand, the bond strength of adhesives was not affected when the restoration procedure was performed before radiation, since radiation cannot alter the structure of the existing hybrid layer [3, 10]. Therefore, this finding may be a potential explanation for the similar bond strengths between the restoration before radiation group and the control group.

This study used only one universal adhesive (Single Bond Universal Adhesive), which could be regarded as a limitation of the study. Further studies should perform bond strength tests with universal adhesives with different compositions. Moreover, long-term data are needed to provide more information.

## Conclusions

Based on the limitations of the current investigation, we concluded that the timing of radiation had an influence on the microtensile bond strength when the restorative procedure was performed after irradiation. When the restoration was performed before irradiation, no significant alteration was detected. In addition, the different adhesive application modes had no effect on the microtensile bond strength.

## Data Availability

The datasets generated and analyzed during the current study are not publicly available but are available from corresponding author on reasonable request.

## Abbreviations

µTBS: Microtensile bond strength
MPa: Megapascal
F-RT: Radiation after restoration
RT-F: Radiation before restoration
ER: Etch-and-rinse mode
SE: Self-etch mode
ANOVA: Analysis of variance
SEM: Scanning electron microscopy
Gy: Gray
MDP: Methacryloyloxydecyl dihydrogen phosphate
HEMA: 2-Hydroxyethyl methacrylate
mW/cm^2^: Milliwatts per square centimeter
MV: Megavolt
°C: Degrees Celsius
h: Hour(s)
min: Minute(s)
s: Second(s)
cm: Centimeter(s)
mm: Millimeter(s)
mm^2^: Square millimeters
HCl: Hydrochloric acid
NaOCl: Sodium hypochlorite
SiC: Silicon carbide
Pt-Pd: Platinum–palladium
Ca/P: Calcium-to-phosphorous ratio
ROS: Reactive oxygen species
